# Improvement of vaginal probiotics *Lactobacillus crispatus* on intrauterine adhesion in mice model and in clinical practice

**DOI:** 10.1186/s12866-023-02823-y

**Published:** 2023-03-22

**Authors:** Fei Wu, Yao Kong, Wenjie Chen, Dingfa Liang, Qin Xiao, Lijuan Hu, Xiao Tan, Jing Wei, Yujuan Liu, Xiaorong Deng, Zhaoxia Liu, Tingtao Chen

**Affiliations:** 1 Department of Obstetrics and Gynecology, The Second Affiliated Hospital of Nanchang University, Nanchang, China; 2 National Engineering Research Center for Bioengineering Drugs and The Technologies, Institute of Translational Medicine, Nanchang University, Nanchang, China; 3 Department of Obstetrics and Gynecology, The Affiliated Hospital of Jiangxi University of Traditional Chinese Medicine, Nanchang, China; 4 Department of Gastrointestinal Surgery, The Second Affiliated Hospital of Nanchang University, Nanchang, China

**Keywords:** Intrauterine adhesion, *Lactobacillus crispatus*, Endometrial injury, High-throughput sequencing, Estrogen

## Abstract

**Background:**

Intrauterine adhesion (IUA) is a frequent acquired endometrial condition, for which there is no effective preventive or treatment. Previous studies have found that vaginal microbiota dysregulation is closely related to endometrial fibrosis and IUA. Therefore, we wondered whether restoration of vaginal microbiota by vaginal administration of *L. crispatus* could prevent endometrial fibrosis and ameliorate IUA.

**Results:**

First, we created a mechanically injured mouse model of IUA and restored the mice’s vaginal microbiota by the addition of *L. crispatus* convolvulus. The observations suggested that intrauterine injections of *L. crispatus* significantly decreased the degree of uterine fibrosis, the levels of IL-1β and TNF-α in blood, and downregulated the TGF-β1/SMADs signaling pathway in IUA mice. A therapy with *L. crispatus* considerably raised the abundance of the helpful bacteria *Lactobacillus* and *Oscillospira* and restored the balance of the vaginal microbiota in IUA mice, according to high-throughput sequencing. Then we conducted a randomized controlled trial to compare the therapeutic effect of *L. crispatus* with estrogen after transcervical resection of adhesion (TCRA). And the results showed that vaginal probiotics had a better potential to prevent intrauterine adhesion than estrogen.

**Conclusions:**

This study confirmed that *L. crispatus* could restore vaginal microbiota after intrauterine surgery, inhibit endometrial fibrosis, and finally play a preventive and therapeutic role in IUA. At the same time, it is a new exploration for the treatment of gynecological diseases with vaginal probiotics.

**Clinical trial registration:**

: http://www.chictr.org.cn/, identifier (ChiCTR1900022522), registration time: 15/04/2019.

**Supplementary Information:**

The online version contains supplementary material available at 10.1186/s12866-023-02823-y.

## Background

Intrauterine adhesion (IUA), a common acquired endometrial disease, typically develops as a result of endometrial damage brought on by trauma, curettage, infection, etc. [1], and its typical clinical signs are amenorrhea, infertility and pelvic pain [2]. Currently, the mainstream treatment for IUA is a combination of treatment based on transcervical resection of adhesion (TCRA) [3] supplemented by intrauterine device (IUD) placement [4], stem cells [5] or estrogen [6]. However, high treatment costs, displacement of the IUD device and re-adhesion after treatment bring heavy psychological and economic burdens to patients [7]. Hence, it is imperative to develop a noninvasive technology that is safe, effective and attractive in order to prevent adhesion from occurring again.

IUA is a prevalent medical disorder that is essentially an inflammatory and fibrotic disease brought on by poor endometrial epithelial regeneration and repair, but the exact mechanism of its occurrence is not clear [8]. It has been demonstrated that NF-κB is a crucial regulator of inflammation-fibrosis [9], which activates and translocates to the nucleus in response to inflammatory factor stimulation, binds specifically to the promoter binding site of TGF-β1 activator, promotes TGF-β1 expression, and activates the TGF-β1/SMADs signaling pathway [10]. The SMADs protein is phosphorylated and binds to the corresponding binding site of NF-κB, which is also phosphorylated, in the nucleus, re-encoding translation and stimulating the production of more cellular inflammatory factors [interleukin-1β, (IL-1β); tumor necrosis factor-α, (TNF-α)], causing persistent damage to cells, promoting fibroblast differentiation, collagen fiber production, and subsequently fibrosis [11, 12].

Moreover, our previous study has confirmed that vaginal microbiota disorder is closely related to IUA [13]. Specifically speaking the quantity of *Lactobacilli* in the vagina of IUA patients decreased from 97% to 45% compared to healthy women of reproductive age, and in 30% (6/20) of them, the presence of *Lactobacilli* in the vagina was barely detectable. Meanwhile, the pathogenic bacteria *Gardnerella* and *Prevotella* sp. were dramatically increased in the vagina of IUA patients. Therefore, we wondered whether restoration of vaginal microbiota by vaginal administration of *L. crispatus* could prevent endometrial fibrosis and ameliorate IUA.

*Lactobacillus crispatus* (*L.crispatus*) is a Gram-positive bacterium that can maintain vaginal microecological health by producing acid, hydrogen peroxide and bacteriocins in the vagina [14], and has good inhibitory effect on common pathogens of vaginitis such as *Candida albicans* and Group B Streptococcal species [15]. In addition, it has good antioxidant properties and can adhere to Hela cells in large numbers and effectively prevent the colonization of Hela cells by opportunistic pathogens [16]. At the same time, it is safe, non-toxic, stable and can be preserved for a long time [17]. To date, little research has examined the connection between the vaginal microbiota and IUA, particularly the possible role of probiotics in the management of IUA. Therefore, we chose *L. crispatus*, which has several advantages, as the probiotics for this study. To explore whether the vaginal probiotic *L. crispatus* can improve IUA, we conducted this study using a combination of basic and clinical science.

## Results

### ***L. crispatus*** prevent and treat intrauterine adhesion in mice

**Fig. 1:**
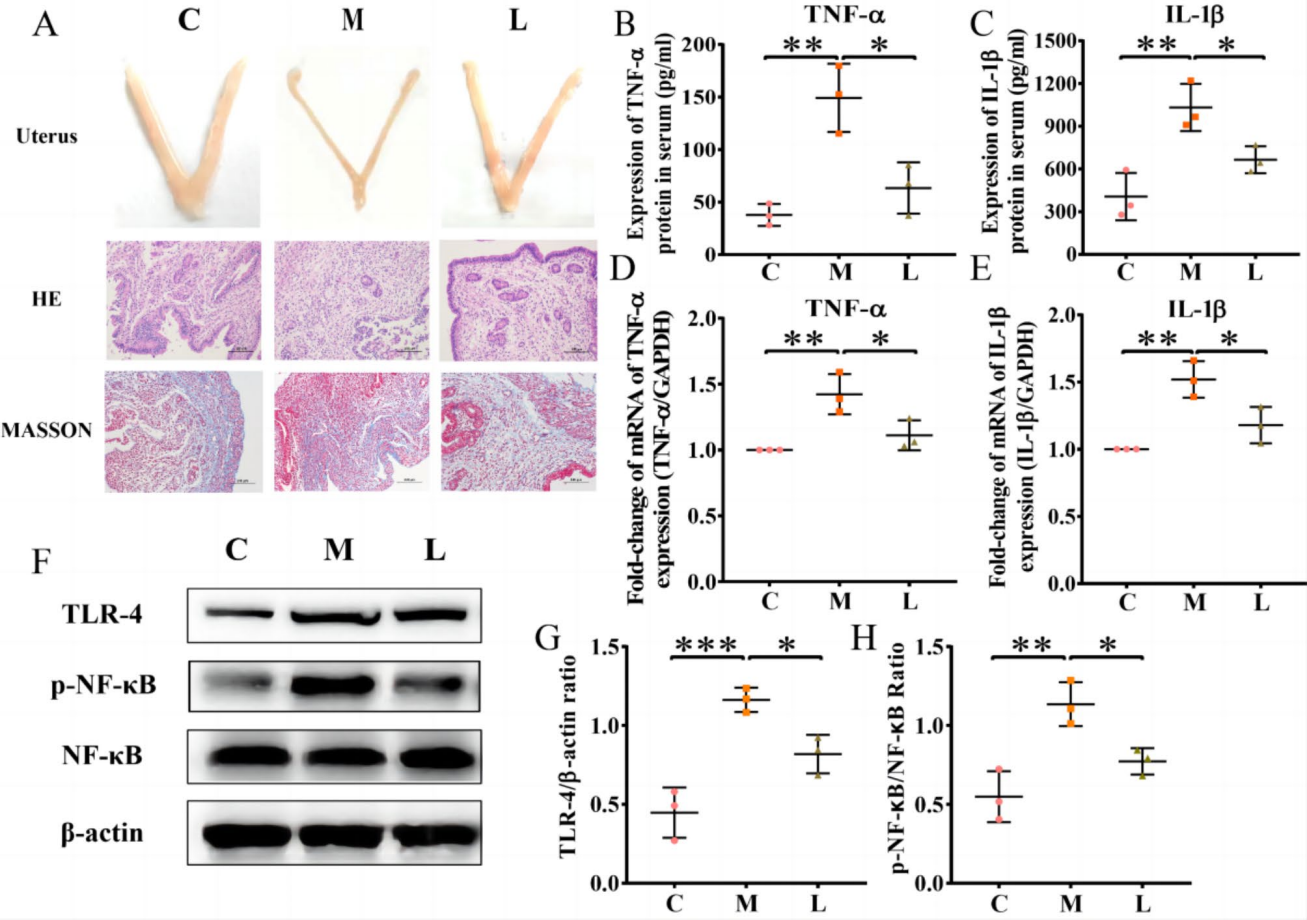
Improvement of fibrosis and inflammation in mice by *L. crispatus*. (**A**)The morphological changes of intrauterine adhesion were observed in the isolated uterine model; He staining was used to observe inflammatory exudation and Masson staining was used to assess collagen fiber deposition in the uterine tissue of intrauterine adhesion mice (magnification: × 100). At the protein level, the effect of *L. crispatus* on the expression of (**B**) TNF-α and (**C**) IL-1β in uterine tissues of mice with intrauterine adhesions. At the gene level, the effect of *L. crispatus* on (**D**) TNF-α and (**E**) IL-1β transcription in uterine tissues of mice with intrauterine adhesion. (**F**) Inflammatory protein expression in uterine tissues of mice with intrauterine adhesion. Effects of *L. crispatus* on inflammation-related (**G**) TLR4 and (**H**) p-NF-κB/NF-κB proteins in uterine tissues of mice with intrauterine adhesion. C group, Control group; M group, Mechanical injury was used to construct a model of intrauterine adhesion; L group was treated with *L. crispatus* for intrauterine adhesion mice. *P < 0.05; **P < 0.01; ***P < 0.001. Western blotting images were cropped to improve the conciseness of the data, and the original images with visible membrane edges can be found in Supplementary Material

In this study, after adaptive feeding, the mice model of intrauterine adhesion was constructed by means of mechanical injury, and then treated with *L. crispatus*. In the end, there were six mice in C and M groups and only five mice in L group. In Fig. 1A, we can observe that the uterus of mice in group M lost elasticity and narrowed uterine cavity compared with control group C. Meanwhile, HE staining and Masson staining additionally demonstrated that the monolayer columnar cell layer on the endometrial surface of mice in group M was broken or even absent, and the fibrous scar tissue gradually replaced or covered the original endometrial group, and the gland structure was small and sparse, accompanied by inflammatory cell infiltration. However, in group L mice treated with *L. crispatus*, uterine morphology improved but did not return to normal levels. The endometrial surface of the mice in group L had a complete and continuous monolayer of columnar cells, endometrial glands also increased, and the infiltration of inflammatory cells and collagen fibers decreased. This was evident from the findings of HE staining and Masson staining, so *L. crispatus* can lessen the harm that mechanical manipulation does to the endometrium.

### ***L. crispatus*** effectively inhibit the production of proinflammatory factors

As TNF-α and IL-1β are the main mediators of local inflammatory response [18], the contents of two proteins in each mouse group’s uterine tissue were detected by ELISA and Q-PCR at the protein and gene levels, respectively, to explore the relationship between IUA and inflammation. As shown in Fig. 1B-E, compared with group C, surgery increased the expression of TNF-α (37.82 to 149.18, P < 0.01) and IL-1β (405.76 to 1031.72, P < 0.01) in the serum in group M, while group L treated with *L. crispatus* decreased TNF-α (149.18 to 63.37, P < 0.05) and IL-1β (1031.72 to 665.03, P < 0.05) expression. Furthermore, the Q-PCR results further demonstrated that intrauterine surgery significantly increased the transcriptional levels of TNF-α (1.00 to 1.42, P < 0.01) and IL-1β (1.00 to 1.52, P < 0.01) in group M, while *L. crispatus* treatment significantly reduced the transcriptional levels of pro-inflammatory factors in group I. However, since activation of signaling pathways is required for the release of inflammatory factors, we used western blotting to further investigate the canonical inflammatory TLR4/NF-κB signaling pathways. In Fig. 1F-H, compared with group C, surgery increased the expression levels of TLR4 (0.45 to 1.16, P < 0.001) and p-NF-κB (0.55 to 1.13, P < 0.01). The opposite trend was expressed in L group mice treated with *L. crispatus.*

### ***L. crispatus*** reduces fibrosis in the models of intrauterine adhesion mice

**Fig. 2:**
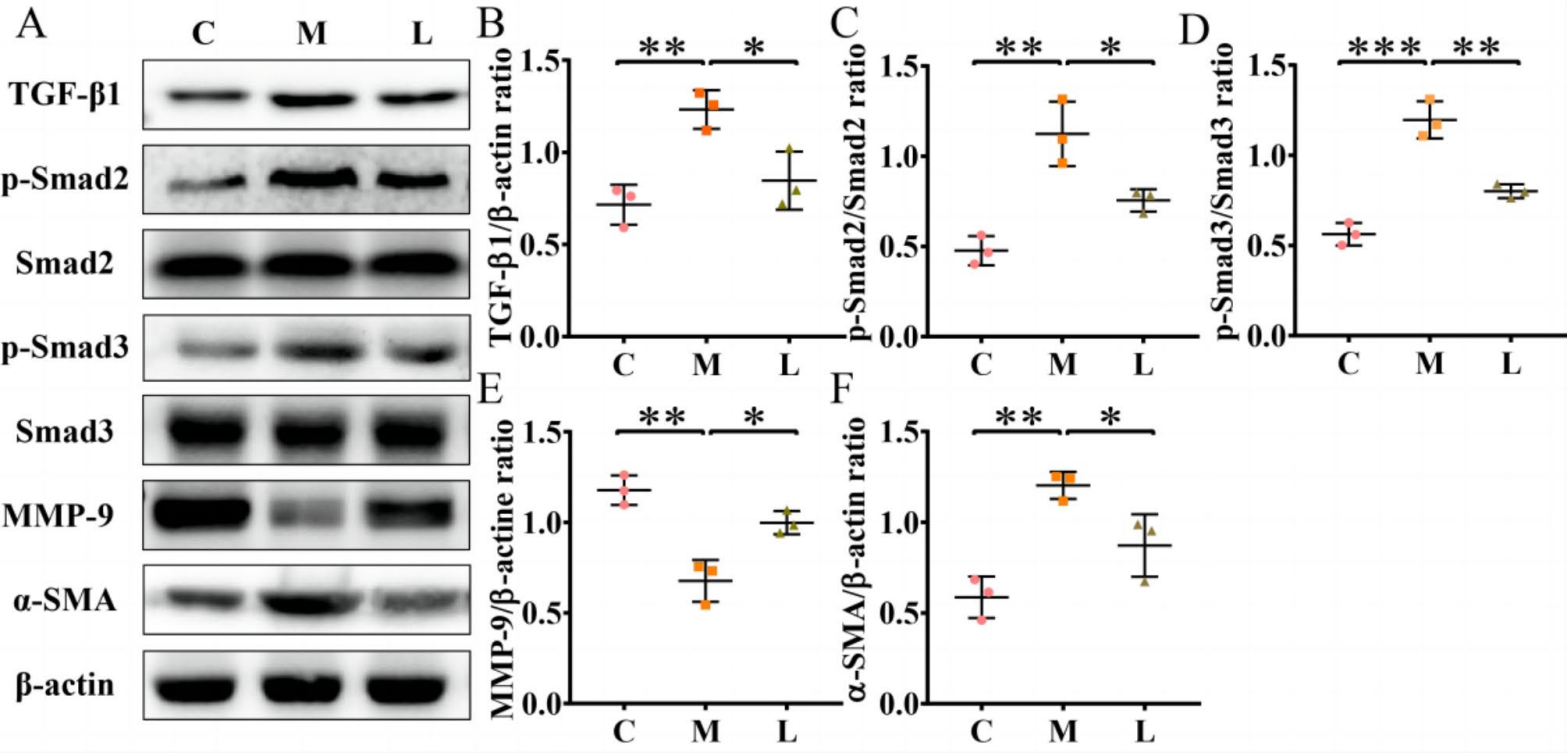
The formation of intrauterine adhesions is closely related to the fibrosis TGF-β1/Smads pathway. (**A**) Expression of fibrosis-related proteins in uterine tissues of intrauterine adhesion mice. Effects of *Lactobacillus crimp* on (**B**) TGF-β1, (**C**) p-smad2 /Smad2, (**D**) p-Smad3/Smad3, (**E**) MMP-9 and (**F**) α-SMA proteins related to fibrosis in uterine tissues of mice with intrauterine adhesion. C group, Control group; M group, Mechanical injury was used to construct a model of intrauterine adhesion; L group was treated with *L. crispatus* for intrauterine adhesion mice. *P < 0.05; **P < 0.01; ***P < 0.001. Western blotting images were cropped to improve the conciseness of the data, and the original images with visible membrane edges can be found in Supplementary Material

Previous studies demonstrated that activation of the TGF-β1/Smads signaling pathway was associated with the appearance of IUA [19, 20], so we used western blotting to assess its expression level at the adhesion site. As shown in Fig. 2A-D, compared with group C, the expression of TGF-β1 (0.72 to 1.23, P < 0.01), p-Smad2 (0.48 to 1.12, P < 0.01) and p-Smad3 (0.56 to 1.20, P < 0.001) increased significantly in group M after surgery. After *L. crispatus* treatment, TGF-β1 (1.23 to 0.85, P < 0.05), p-Smad2 (1.12 to 0.75, P < 0.05) and p-Smad3 (1.20 to 0.80, P < 0.01) expression levels were restored in group L. Therefore, we studied the expression of Matrix metalloproteinase-9 (MMP-9) and α-Smooth muscle actin (α-SMA) in each group, and found that intrauterine surgery did down-regulate MMP-9 (Fig. 2E), which was significantly improved after *L. crispatus* treatment, while α-SMA had the opposite result (Fig. 2F).

### ***L. crispatus*** improved the vaginal microbiota of mice with IUA

**Fig. 3:**
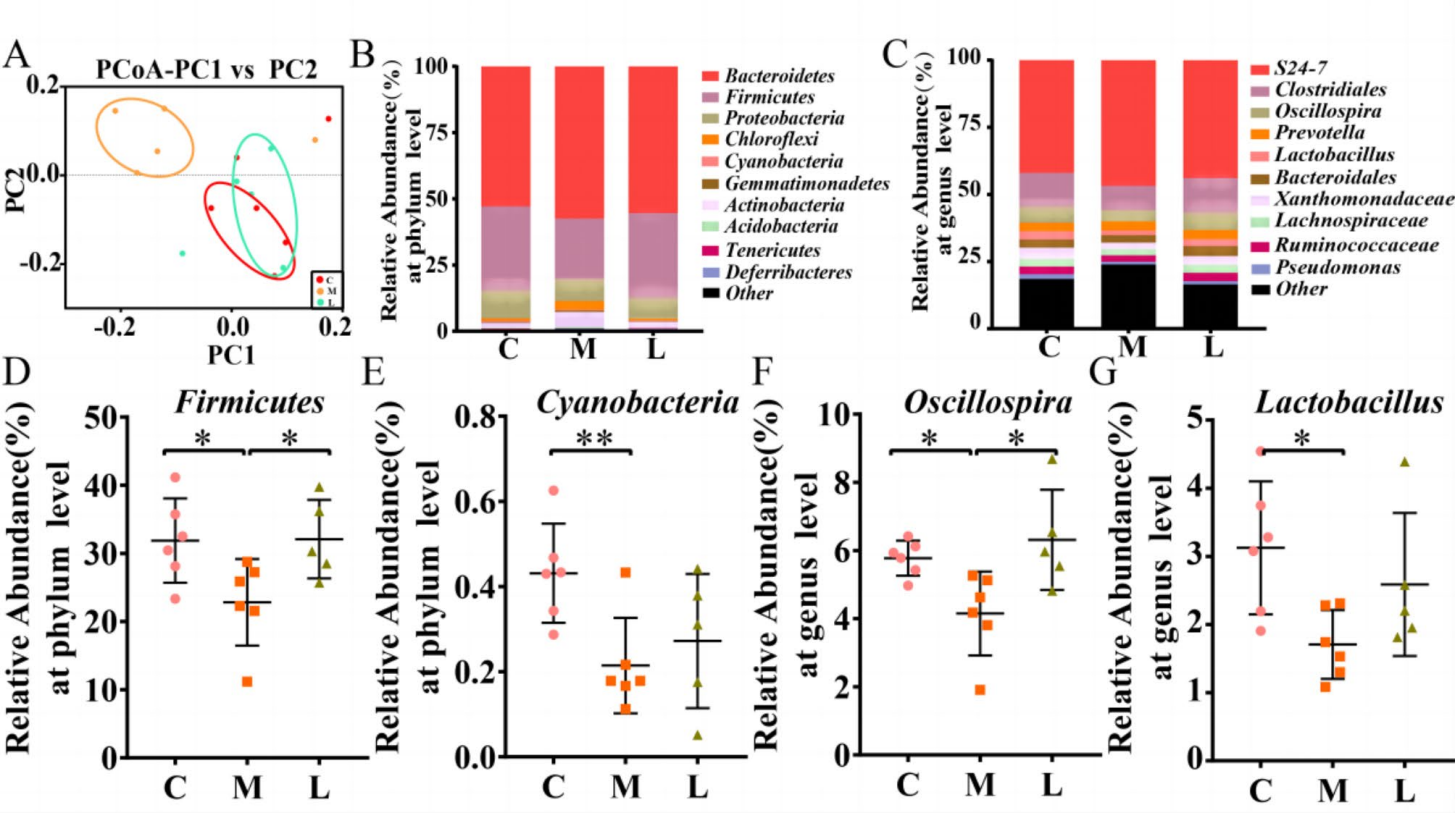
Effect of *L. crispatus* involved on vaginal microbiota in mice with intrauterine adhesion. Evaluation of the effect of *L. crispatus* on vaginal microbiota of intrauterine adhesion mice using (**A**) the PCoA of the β diversity index, the relative abundance (**B**) at the phylum level and (**C**) at the genus level. At the phylum level, the effects of *L. crispatus* on (**D**) *Firmicutes* and (**E**) *Cyanobacteria* were evaluated. At the genus level, the effects of *L. crispatus* on (**F**) *Oscillospira* and (**G**) *Lactobacillus* were evaluated. C group, Control group; M group, Mechanical injury was used to construct a model of intrauterine adhesion; L group was treated with *L. crispatus* for intrauterine adhesion mice. ns, *P* > 0.05; *P < 0.05; **P < 0.01

In the previous study, we found that there were significant differences in vaginal microbiota of patients with IUA compared with normal women. Therefore, we used high-throughput sequencing technology to explore whether the vaginal microbiota of mice in each group was different. According to the PCoA results (Fig. 3A), most of the points in group L are close to group C, while the points in group M are scattered away from group C. Subsequently, we compared the differences of the top 10 microbiota in the vaginal microbiota of each group at phylum level and genus level (Fig. 3B-C). In Fig. 3D-E, compared with C group, the richness of *Firmicutes* in M group (31.88 to 22.81%, P < 0.05) decreased to a certain extent, while after treatment with *L. crispatus*, this bacteria in L group (22.81 to 32.09%, P < 0.05) increased compared with those before treatment. However, *Cyanobacteria spp.* showed the opposite trend. Intrauterine operation could reduce its richness (0.43 to 0.21%, P < 0.01), but after treatment with *L. crispatus*, its richness increased (0.21 to 0.27%, P > 0.05). In Fig. 3F-G shows the results of vaginal microbiota at genus level. The abundance of *Oscillospira* in group M was higher than that in group C (5.78 to 4.15%, P < 0.05). This trend was reversed with the supplementation with *L. crispatus*, with a significant decrease in *Oscillospira* abundance in the L group (4.15 to 6.31%, P < 0.05) when compared to group M. However, the content of *Lactobacillus* (3.12 to 1.71%, P < 0.05) in group M was reduced by the operation, but the abundance of *Lactobacillus* (1.71 to 2.59%, P > 0.05) in group L increased obviously after *L. crispatus* treatment.

## Clinical investigation of patients with IUA

**Fig. 4:**
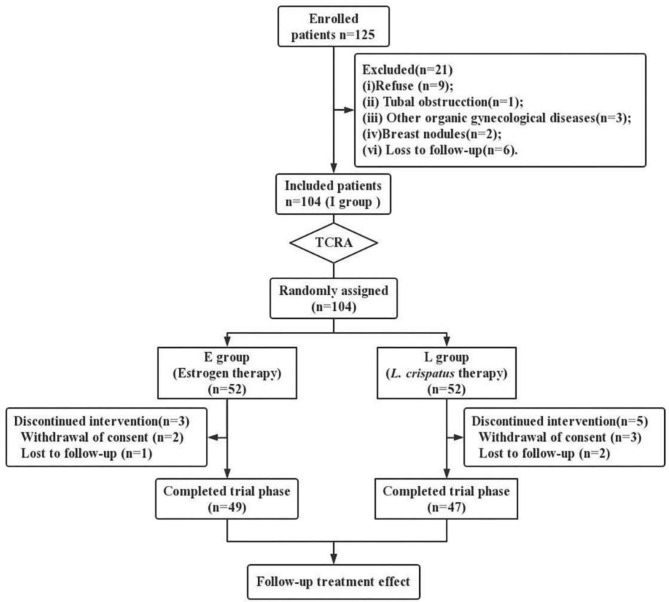
The flow chart of a clinical trial

A total of 125 people were evaluated for eligibility from January 2020 to December 2021; of those, 104 were assigned at random and used in the study, 52 in both the E and L groups. During this process, 3 participants in group E (2 withdrawal of consent, 1 lost to follow-up) and 5 participants in group L (3 withdrawal of consent, 2 lost to follow-up) failed to complete the study (Fig. 4). In addition, the incidence of CD38 (+) and CD138 (+) in group I was 27.08% (14 + 12/49 + 47) after routine pathological examination of endometrium after TCRA. The participants in E group and L group were graded as mild, moderate and severe according to the Chinese intrauterine adhesion diagnostic grading criteria [21], and no discernible distinction could be made between the two groups. The thickness of the endometrium before and after treatment, and the cure rate and recurrence rate after TCRA were statistically compared between the two groups (Table 1). Among them, 17 patients (34.69%) in group E had postoperative recurrence, while only 8 patients (17.02%) in group L, showed P < 0.05 by X^2^ test.

**Table 1:** Comparison of the efficacy of E group and L group after operation for IUA

Variables	E group	L group	P value
**Number**	49	47	
**Age**	29.76 ± 0.5568	29.87 ± 0.5942	0.8857
**Degree of adhesion**			
**Mild adhesion**	6 (12.24%)	7 (14.89%)	
**Moderate adhesion**	31 (63.27%)	29 (61.70%)	
**Severe adhesion**	12 (24.49%)	11 (23.41%)	
**CD38(+) and CD138(+)**	14 (28.57%)	12 (25.53%)	
**Endometrial thickness (mm)** ^**a**^			
**Pre-operation**	4.955 ± 0.2185	5.155 ± 0.2491	0.9437
**Post operation**	7.178 ± 0.2993	6.291 ± 0.2352	0.0667
**Postoperative effect** ^**b**^			
**Cure**	32 (65.31%)	39 (82.98%)	0.0486
**Recurrence**	17 (34.69%)	8 (17.02%)	

^**a**^Mean ± SD, t-test

^**b**^Number (percentage), X^2^ test

### *L. crispatus* can improve IUA vaginal microbiota

**Fig. 5:**
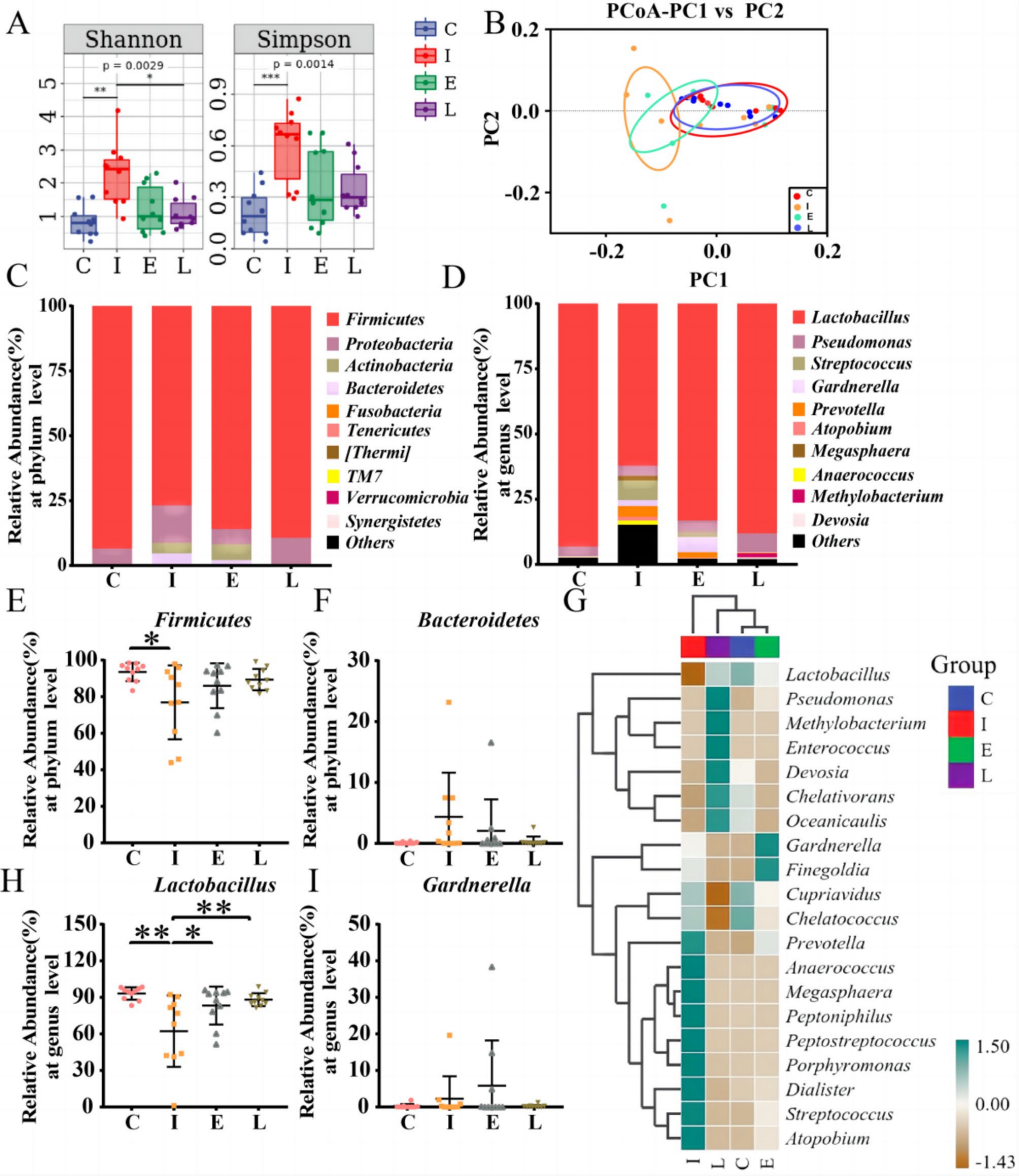
The effect of *L. crispatus* and Estrogen on vaginal microbiota in patients after TCRA. Evaluation of the effect of *L. crispatus* and Estrogen on vaginal microbiota of TCRA using: (**A**) the Shannon index and the Simpson index; (**B**) the PCoA of the β diversity index; the relative abundance (**C**) at the phylum level and (**D**) at the genus level. At the phylum level, the effects of *L. crispatus* and Estrogen on (**E**) *Firmicutes* and (**F**) *Bacteroidetes* were evaluated. At the genus level, the effects of *L. crispatus* on (**H**) *Lactobacillus* and (**I**) *Gardnerella* were evaluated. (**G**) the cluster heat map of IUA before and after treatment compared with healthy women. C group, the healthy female control group; I group, patients with untreated IUA; E group was treated with Estrogen after TCRA surgery; L group was was treated with *L. crispatus* after TCRA surgery. *P < 0.05; **P < 0.01 We allow visitors to reproduce images with permission and/or credit

Finally, 10 vaginal secretion samples were selected in the group C, I, E and L, a total of 40 samples were used for high-throughput sequencing. In α-diversity, there are significant differences in Shannon (P < 0.01) and Simpson (P < 0.005) (Fig. 5A). Principal coordinate analysis (PCoA) of the β-diversity data revealed that the microbiological diversity of each group varied (Fig. 5B). An aggregated heat map was also produced for each group by association of the 20 vaginal microbes with the highest average abundance at the genus level (Fig. 5G). According to the findings, group C had the highest and group I had the lowest relative abundance of the beneficial bacterium *Lactobacillus*. Moreover, in Group I, the content of the following bacteria is relatively high, such as *Prevotella*, *Anaerococcus*, *Megasphaera*, etc. In group E, there was a greater relative abundance of *Gardnerella* and *Finegoldia*. But in the L group, the relative content of *Pseudomonas*, *Methylobacterium*, *Enterococcus*, *Devosia* and other bacteria was higher. The intrauterine adhesions before and after treatment were compared with vaginal microbiota in healthy women at phylum and genus levels. Significant changes were found in the composition of each classification and the taxonomic composition of vaginal microbiota in each group was compared (Fig. 5C-D). At the phylum level (Fig. 5E-F), compared with group C, the beneficial bacteria *Firmicutes* (93.52 to 76.88%, P < 0.05) in patients with IUA decreased, while *Bacteroidetes* (0.09 to 4.37%, P > 0.05) increased. After estrogen treatment, *Firmicutes* (76.88 to 85.99%, P > 0.05) increased, while *Bacteroidetes* (4.37 to 2.05%) decreased. This trend was even more pronounced after treatment with *L. crispatus* (76.88 to 89.35%, 4.37 to 0.31%). At the genus level (Fig. 5H-I), *Lactobacillus* was significantly reduced in group I (93.14 to 62.19%, P < 0.01), while *Gardnerella* (0.24 to 2.27%, P > 0.05) was increasing. After treatment, the content of *Lactobacillus* in Group E (62.19 to 83.18%, P < 0.05) and group L (62.19 to 88.11%, P < 0.01) increased. The relative levels of *Gardnerella* decreased in groups E (2.27 to 5.83%, P > 0.05) and L (2.27 to 0.19%, P > 0.05).

## Discussion

A combination of TCRA supplemented with IUD [22], balloon [23], intrauterine anti-adhesive [24] and amniotic membrane [25] are the primary methods of treating IUA. Postoperative dysbiosis, overgrowth of pathogenic bacteria and displacement of IUD will lead to serious physiological disorders and disruption of microecological balance, which in turn will affect the patient’s postoperative recovery and prognosis [26].

Nowadays, the antagonistic and synergistic effects between vaginal microbiota are significant in keeping the female reproductive system in good condition [27]. One or a few *Lactobacilli*, such as *Lactobacillus iners, L. crispatus, Lactobacillus jensenii, and Lactobacillus gasseri*, which exhibit relatively low microbial diversity, predominate in the healthy reproductive women’ vaginal microbiota [28]. Studies have reported that surgical stimulation, use of prophylactic antibiotics, and irrigation of large amounts of normal saline may destroy reproductive tract microbes balance, leading to retrograde infection of pathogenic bacteria in the vagina and inflammation of the uterine cavity [16–29]. At present, the clinical application of vaginal probiotics is mainly for the treatment of different forms of vaginitis, and few studies have investigated the possible role of probiotics in the treatment of IUA [30]. So this study aims to investigate the therapeutic effect and mechanism of vaginal probiotics on IUA through animal and clinical experiments.

It is well known that the constant stimulation of infection and inflammation impairs the repair of endometrial basal layer damage, leading to intrauterine inflammation and fibrosis, and ultimately to IUA [31]. In animal experiments, we evaluated the treatment effect from mouse uterine morphology, HE staining and Masson staining. And ELISA and Q-PCR were employed to identify the expression of mouse serum IL-1β and TNF-α pro-inflammatory factors, because inflammation has a substantial impact on fibrosis [32]. Then, western blot analysis was used to determine the protein expression of the inflammatory signaling pathways (TLR4, p-NF-κB and NF-κB) and the fibrosis signaling pathways (TGF-β1, p-Smad2, Smad2, p-Smad3, Smad3, MMP-9, α-SMA). The results showed that *L. crispatus* considerably reduced the uterine injury’s inflammatory and fibrotic reactions and postponed the occurrence of IUA. In the process of further research on the mechanism of action, we discovered that probiotics significantly decreased the pro-inflammatory factors IL-1β and TNF-α and downregulated TLR4/NF-κB Inflammatory Signaling pathways. In IUA tissue, studies have demonstrated that NF-κB is considerably up-regulated and activated, promoting the production of IL-1β and TNF-α, two chemicals that cause inflammation [11]. Tissue fibrosis occurs as a result of these pro-inflammatory chemicals’ actions on fibroblasts [12]. Inferring that probiotics prevent IUA by lowering the inflammatory response, *L. crispatus* therapy decreased the expression of essential proteins in the TLR4/NF-κB signaling pathway.

The purpose of IUA treatment is to reduce endometrial fibrosis and boost endometrial regeneration. TGF-β1 and MMP-9 are two profibrotic and antifibrotic cytokines whose interactions control the damaged endometrial healing process [33]. Smad2 and Smad3 are two significant downstream regulators that encourage TGF-β1 mediated tissue fibrosis [34], and their phosphorylation functions play an important role in avoiding TGF-β1 mediated fibrosis [35]. The degree to which α-SMA, a marker of myofibroblasts, is expressed reflects the degree of fibrosis production [36]. MMP-9, a downstream target gene of TGF-β1, is considered as an anti-fibrosis factor due to its ability to degrade and reshape extracellular matrix (ECM) [37]. This study confirmed that surgery increased fibrosis in model mice, resulting in up-regulation of α-SMA and down-regulation of MMP-9. However, the use of *L. crispatus* significantly reduced the essential proteins linked to the TGF-β1/Smads pathway (TGF-β1, p-Smad2, p-Smad3 and α-SMA), while considerably boosted the level of MMP-9.

In high-throughput sequencing results, the composition and diversity of vaginal microflora in mice with IUA were significantly changed, as shown by PCoA in Fig. 3A. So, we may infer that this population’s microbiota is unbalanced. But this imbalance in vaginal microbiota improved after *L. crispatus* intervention, consistent with previous studies [38]. Vaginal dysbiosis promotes colonization of the vagina by pathogens and leads to the formation of bacterial biofilms and an increased risk of vaginal infection, implying the occurrence and recurrence of chronic diseases [39]. The results of the microbiota at the genus level showed that there was a significant decrease in the abundance of I group *Oscillospira* and *Lactobacillu*, and the treatments with *L. crispatus* showed a large increase in the abundance of both species, and made the composition of vaginal microorganisms after treatment more similar to normal levels. *Oscillospira*, as one of the producers of butyrate [40], can be considered as a beneficial bacterium of the vagina to maintain the health of the female vagina, mainly because butyrate promotes mucosal repair and functional recovery, inhibits the formation of inflammatory cytokines, and has anti-inflammatory effects [41]. Moreover, *Lactobacillus* can ferment sugars to produce lactic acid, maintaining the acidic environment of the vagina, which is thought to be highly protective against infection or vaginal colonization by pathogens and non-native microorganisms. In addition, it can secrete bacteriocins and other antimicrobial factors to inhibit or kill pathogenic microorganisms [42]. So, *Lactobacillus* plays an important role in the maintenance of vaginal microbial homeostasis. There have been studies in the treatment of human papilloma virus (HPV) [43] and bacterial vaginosis (BV) [30] by vaginal administration of *Lactobacillus* to restore vaginal microbiota composition. Therefore, the homeostasis of women’s vaginal microbes plays a significant role in women’s health [44, 45]. This study suggests that the formation of IUA is related to the imbalance of vaginal microbiota. Vaginal administration of *L. crispatus* to improve the imbalance of vaginal flora has therapeutic effect on IUA mice.

In the clinical investigation, 27.08% of patients with IUA had endometritis, which were positive for CD38 and CD138, which was consistent with previous studies on the etiology of intrauterine adhesion [46]. Therefore, we speculated that the cervical mucus plug may be destroyed during intrauterine surgery, causing the spread of pathogenic bacteria to the uterine cavity and increasing the risk of intrauterine infection, leading to the occurrence of intrauterine adhesions and endometritis [47, 48].

After TCRA, by comparing with estrogen, we found that the effect of estrogen in promoting endometrial growth is better than that of *L. crispatus*, but the recurrence rate after TCRA can reach 34.69%. This confirmed that postoperative estrogen use promotes endometrial growth and inhibits endometrial fibrosis [33]. However, increased estrogen levels are conducive to the dominance of *Lactobacillus* in vaginal microbiota, but the incidence of vulvovaginal candidiasis (VVC) is also increased [49]. Under the influence of female high estrogen, the accumulation of glycogen in the vaginal epithelial cells increases, the lactic acid is increased by the decomposition of *Lactobacillus*, the pH value in the vagina decreases, and the microecological balance in the vagina is destroyed, which is conducive to the survival of anaerobic pathogens suitable for an acidic environment, also contribute to the adhesion of pathogens [50]. In addition, under the action of estrogen, the congestion, edema and permeability of the vaginal mucosa increase, which may make the vaginal mucosa more vulnerable to injury than before treatment, thus making it more prone to vaginal infection [51], and eventually leading to the occurrence of IUA [2]. Meanwhile, long-term estrogen therapy may affect women’s health, including fertility, diabetes, obesity and cancer [52].

Although *L. crispatus* was inferior to estrogen therapy in promoting endometrial growth, its cure rate was as high as 82.98% (P < 0.05). High-throughput results showed significant changes in vaginal microbiota composition between patients with IUA and healthy women. Treatment with *L. crispatus* improved vaginal microbiota more than treatment with estrogen. Not only was α-diversity statistically significant, but PCoA also confirmed this result. At the genus level. *Lactobacillus* in patients with Intrauterine adhesion was significantly reduced, but after estrogen therapy, especially after *L. crispatus* treatment, *Lactobacillus* was close to the level of normal women. The opposite trend was observed in pathogenic *Gardnerella*. Therefore, we infer that vaginal microbiota dysbiosis exacerbates the development of IUA, this is consistent with our previous research results [13]. Furthermore, any imbalance in the vaginal microbiota can lead to vaginal infections, such as bacterial vaginitis (BV), aerobic vaginitis (AV), atrophic vaginitis, candida vaginitis, and trichomonad vaginitis [53]. Studies have shown that the reduction of vaginal *L. crispatus* may increase the risk of HIV [42] and HPV [43] infection, as well as the incidence of BV [30], polycystic ovary syndrome (PCOS) [52] and endometriosis (EMS) [54]. Therefore, vaginal administration of *L. crispatus* can not only reduce the recurrence rate after Intrauterine adhesion, but also effectively prevent and treat other gynecological diseases and improve fertility.

This study confirmed that the supplementation of *L. crispatus* can promote early postoperative recovery of patients with intrauterine adhesions by restoring the vaginal microbial balance and inhibiting uterine inflammation and fibrosis. However, some limitations should be taken note. We know that the vaginal microbiota is a complex biological system, and we did not further purify the specific components of *L. crispatus* that play a role in the treatment of intrauterine adhesion. Additionally, the study included a small volunteer base, which reduced the validity of the statistical analysis. And we did not follow up on the pregnancy and reproductive status of the patients after treatment.

## Conclusions

This study confirmed that *L. crispatus* could promote early postoperative recovery in patients with IUA by restoring vaginal microbial balance and inhibiting uterine inflammation and fibrosis. Although vaginal probiotics have been explored in the treatment of gynecological diseases, large sample and multi-center clinical data are still needed to confirm their efficacy in the treatment of luminal adhesion.

## Methods

### Animal models and treatments

A total of 32 female adult SPF BALB/C mice (HUNAN SJA Laboratory animal co., LTD), weighing 22–26 g and aged 6–8 weeks, were employed in this experiment. The mice were placed in a clean, cozy, air-conditioned space with unrestricted access to food and drink. The temperature, lighting, noise, ventilation and other conditions of the observation room are controlled within the specified range. After one week of adaptation, 32 mice with similar body weight and strong adaptability were selected and divided into 3 groups, including the control group (C, n = 8) and 2 experimental groups (n = 12 per group). These experimental groups were divided into M (model of IUA) and L (IUA + *L. crispatus*) groups. All animal procedures were completed at the Institute of Translational Medicine of Nanchang University after approval by the Ethical Committee of Nanchang Royo Biotech Co., Ltd (reference number RYE2019121702).

After adaptation, the IUA model of mice in all experimental groups was established by using the same mechanical injury method as Yang Huan’s [55] according to the clinical surgical requirements. Briefly, the mice were anesthetized by intraperitoneal injection of 1% sodium pentobarbital (100 mg/kg; Cat# B1202-005; Fluka), and the abdominal cavity was opened to expose the uterus after disinfection and sterile surgical towel covering. After preparing a 2 mm transverse incision in the upper part of the uterus, a curettage was used to create a 1.5-2.0 cm endometrial lesion without puncturing the uterine wall. After suturing the uterus and surgical incisions, each mouse was fed separately for 2 weeks to recover. Compared with the model group (M group), the L group was given 1 × 10^8^CFU/mL/day *L. crispatus* (*L. crispatus*, Lcr-MH175, number CGMCC 15,938, HarbinMeihua Biotechnology Co., Ltd., Harbin, Heilongjiang, China) every night using absorbable gelatin sponge in their vaginas for consecutive 2 weeks after surgery. Vaginal secretions [56] from each group were collected consecutively for 5 days prior to euthanasia, and sufficient samples were obtained for high-throughput sequencing. At the end of the two weeks, the mice were euthanized and their venous blood, uterine tissue, and vaginal tissue were collected and appropriately kept for later investigations.

### Histological analysis

Histological examination was carried out in accordance with earlier studies [37]. Prior to being cut into 6 μm transverse slices, mouse uterine tissue was first fixed with 10% paraformaldehyde, dried in a graded ethanol solution, and embedded in paraffin. After HE and Masson stains were applied to all of the slices, under a microscope, the pathogenic alterations were seen.

### Cytokine assays

Mouse serum was obtained by centrifugation at 1000x g for 20 min at 4 ° C, after which serum cytokine concentrations were measured using ELISA kits for IL-1β(Cat#SEA563Mu; mouse; Cloud-Clone Crop; sensitivity range: 15.6–1,000 pg/mL; concentration range used for generating calibration curves: 1,000, 500, 250, 125, 62.5, 31.2, 15.6 and 0 pg/mL) and TNF-a (Cat# SEA133Mu; mouse; Cloud-Clone Crop; sensitivity range: 15.6–1,000 pg/mL; concentrations used for generating calibration curves: 1,000, 500, 250, 125, 62.5, 31.2, 15.6 and 0 pg/mL), according to the manufacturer’s instructions.

### Q-PCR assays

As previously reported [57], Q-PCR was carried out according to the manufacturer’s instructions. Using a high purity total RNA rapid extraction kit (Gibco BRL; Thermo Fisher Scientific), total RNA was isolated from mouse uterine tissue. In addition, the purity and integrity of RNA were evaluated using a NanoDrop 2000 spectrophotometer (Thermo Fisher Scientific, Inc.). Genomic DNA was then removed at 42°C for 2 min, reverse transcribed at 37°C for 15 min, and reverse transcriptase inactivated at 85°C for 5 s to synthesize cDNA. Next, Quantitative real-time PCR was performed using a 7500HT fast real-time PCR system (ABI; Thermo Fisher Scientifc, Inc.). Next, Quantitative real-time PCR was performed using a 7500HT fast real-time PCR system (ABI; Thermo Fisher Scientifc, Inc.). Forty cycles at 95˚C for 30 sec and 60˚C for 30 sec were conducted, preceded by 1 min at 95˚C. Then use the 2^–ΔΔ^ Ct comparison method to calculate the mRNA level, and finally use the GAPDH mRNA expression normalization analysis. The following primers were used in reference to the previous literature [58]: TNFα sense, 5’-GTGGAACTGGCAGAAGAGGCA-3’ and antisense, 5’AGAGGGAGGCCATTTGGGAAC-3’; IL-1β sense, 5’-GTGTCTTTCCCGTGGACCTTC-3’ and antisense, 5’TCATCGAGCTGTAGTGC-3’.

### Western blot analysis

Standard methods were used to perform Western blotting [58]. To put it simply, the proteins from each group’s uterus were isolated, purified to a specific level of purity. Polyacrylamide gel electrophoresis (SDS-PAGE) was used to separate equal amounts of proteins, which were then transferred to a polyvinylidene fluoride membrane. Following a 2-hour soak in 5% skim milk with Tris-buffered saline and TBST to inhibit nonspecific binding sites, the membrane was incubated with the following primary antibodies overnight at 4 °C. Mouse anti TLR4 (Santa Cruz Biotechnology), rabbit anti-NF-κB (ProteinTech Group), rabbit anti-phosphorylated-NF-kB (p-NF-kB; Abcam), rabbit anti-TGF-β1 (ProteinTech Group), rabbit anti p-Smad2 (Cell Signaling Technology), rabbit anti-Smad2 (ABclonal), rabbit anti-p-Smad3 (Cell Signaling Technology), rabbit anti-Smad3 (ABclonal), rabbit anti-MMP-9 (ABclonal) and rabbit anti-β-actin (Cell Signaling Technology). After being washed three times with TBST for ten minutes each, the membrane was incubated for an hour at 25 °C with goat anti-rabbit secondary antibody (ProteinTech Group) or goat anti-mouse secondary antibody (ProteinTech Group) at a dilution of 1:5000. Enhanced chemiluminescence agents were utilized to determine the protein concentrations, and Image J gel analysis software was used to quantify the intensities. The alterations of associated proteins were then calculated and examined using the internal control.

### Patient samples and treatments

January 1, 2019 to June 30, 2020, a total of 125 patients diagnosed with IUA by hysteroscopy in the Jiangxi and Jiujiang maternal and child health hospital in China were enrolled. Patients with untreated IUA were included in I group, and the inclusion criteria were(i) age ranged from 18 to 40; (ii) IUA was the hysteroscopy’s underlying diagnostic in the outpatient setting; (iii) endocrines and ovulation were normal. The exclusion criteria included: (i) fallopian tube problems include hydrosalpinx or obstruction; (ii) other organic gynecological diseases and other basic diseases related to hormones; and (iii) refuse, irregular use of medication and loss to follow-up. Participants were randomly divided into E group (Estrogen therapy) and L group (*L. crispatus* therapy). All patients underwent a series of medical evaluations prior to participation, including a medical history review, physical examination, blood work and transvaginal ultrasound. For the ease of a later examination, the endometrium and vaginal secretions were also collected and cryopreserved in the specimen tube. Samples of vaginal discharge and clinical data were obtained from the patients. The clinical experiment was approved by the Institutional Review Board (IRB) of the Second Affiliated Hospital of Nanchang University, registered in the Chinese Clinical Trial Registry (registration number: ChiCTR1900022522). And it was conducted according to the ethical principles of the Declaration of Helsinki and Good Clinical Practice guidelines. All participants also provided a signed written informed consent.

Groups E or L were randomly assigned to each participant, and adhesions should be separated by hysteroscopy after completion of relevant examinations and a small amount of endometrium was collected during the operation for routine pathological examination. The most widely utilized hormone therapy was employed to treat the patients in group E [6] : Oral estradiol valerate or its equivalent for 21 days, with a daily dose of 4 mg, and 10mgprogesterone acetate or its equivalent was added after 21 days for one week. However, patients in group L used vaginal capsules made of *L. crispatus* (Lcr-MH175, number CGMCC 15,938, HarbinMeihua Biotechnology Co., Ltd., Harbin, Heilongjiang, China) lyophilized powder (1 × 10^8^ CFU/ granule) every night for 2 weeks after surgery. All patients came to the hospital three days after their next clean menstruation to check whether they adhered again, and their vaginal secretions were collected for later high-throughput sequencing. To more accurately assess the alterations in vaginal microbiota in intrauterine adhesion patients before and after therapy, not only the vaginal secretions of 10 people in each group of groups I, E and L were randomly selected, but also the vaginal secretions of 10 healthy women meeting the following conditions were selected as control group C. These included: (i) age 18–40 years with normal menstrual cycle (28 ± 7 days); (ii) no obvious abnormalities were found in vaginal discharge, cervical cancer screening and gynecological ultrasound examination; (iii) no history of other chronic underlying diseases or surgical procedures.

### DNA extraction and highthroughput sequencing

Vaginal secretions were collected from mice and patients in each group, and bacterial genomic DNA was extracted according to the instructions for use of DNA kit from Tiangen Biotech Co., Ltd. The nano drop spectrophotometer (NanoDrop; Thermo Fisher Scientifc, Inc.) to measure the concentration and quality of DNA. The 16 S ribosomal DNA (rDNA) V4 region was amplified using primers (F, AYTGGGYDTAAAGNG; R, TACNVGGGTATCTAATCC) in each sample, and the Q-PCR products were sequenced on the IlluminaHiSeq 2000 platform (GenBank accession number PRJNA 882,985 and PRJNA 883,005). Amplicon generation and sequencing were completed in PersonalbioCo., Ltd. (Shanghai, China).

### Statistical analysis

QIIME (v1.8.0, http://qiime.org/), FLASH (v. 1.2.7, http://ccb.jhu.edu/software/FLASH/), the UCLUST software package and R software were used to analyze the high-throughput sequencing data and evaluate the diversity both within and between samples. Prism software (version 7.0; GraphPad Software, Inc.) was used to evaluate all the data. The information was presented as mean and standard deviation (SD). The one-way analysis of variance (ANOVA), Student’s t test, and X^2^ test were all used to determine statistical significance. Statistical significance is thought to be indicated by a P value of 0.05. The data were presented as mean SD for continuous variables like age that meet the parameters for a normal distribution. If not, quartiles are utilized to express them.

## Electronic supplementary material

Below is the link to the electronic supplementary material.

Supplementary Material 1

## Data Availability

Database bacteria for high-throughput sequencing in animal and clinical trials are available at the National Center for Biotechnology Information (NCBI), and accession number can be found below: PRJNA 882,985 and PRJNA 883,005, respectively ( https://www.ncbi.nlm.nih.gov/bioproject/PRJNA882985 and https://www.ncbi.nlm.nih.gov/bioproject/PRJNA883005 ).
