# Supplementary material for: Improvement of vaginal probiotics Lactobacillus crispatus on intrauterine adhesion in mice model and in clinical practice
Source: BMC Microbiol. 2023 Mar 22;23:78. doi: 10.1186/s12866-023-02823-y (PMC10032012; doi:10.1186/s12866-023-02823-y)
Supplement: Supplementary file 1 — Supplementary Material 1 [file 12866_2023_2823_MOESM1_ESM.pdf]

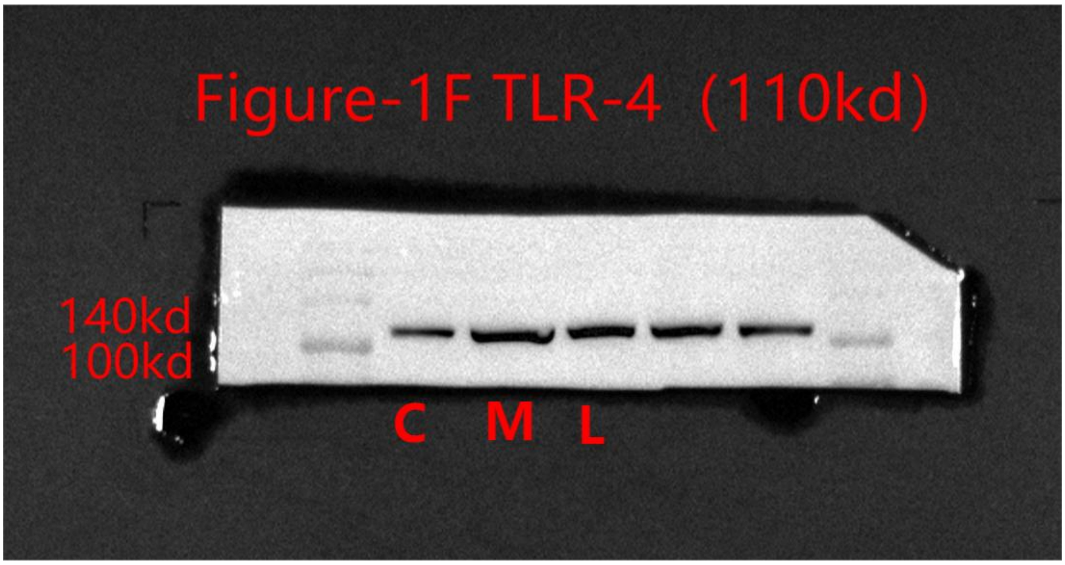
Figure-1F TLR-4 (110kd)
140kd
100kd
C M L

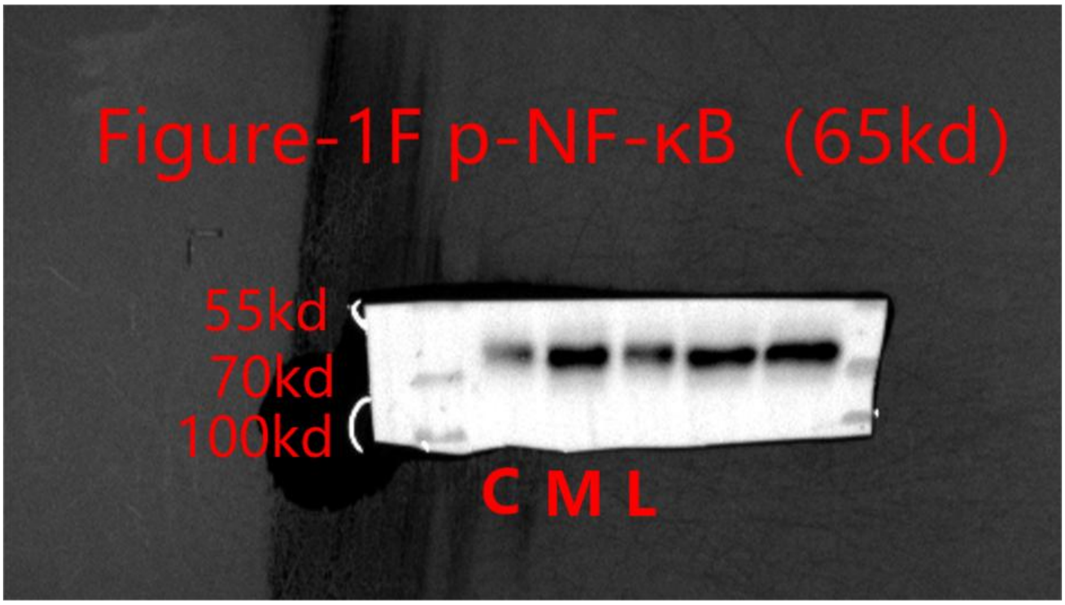
Figure-1F p-NF-κB (65kd)
55kd
70kd
100kd
C M L

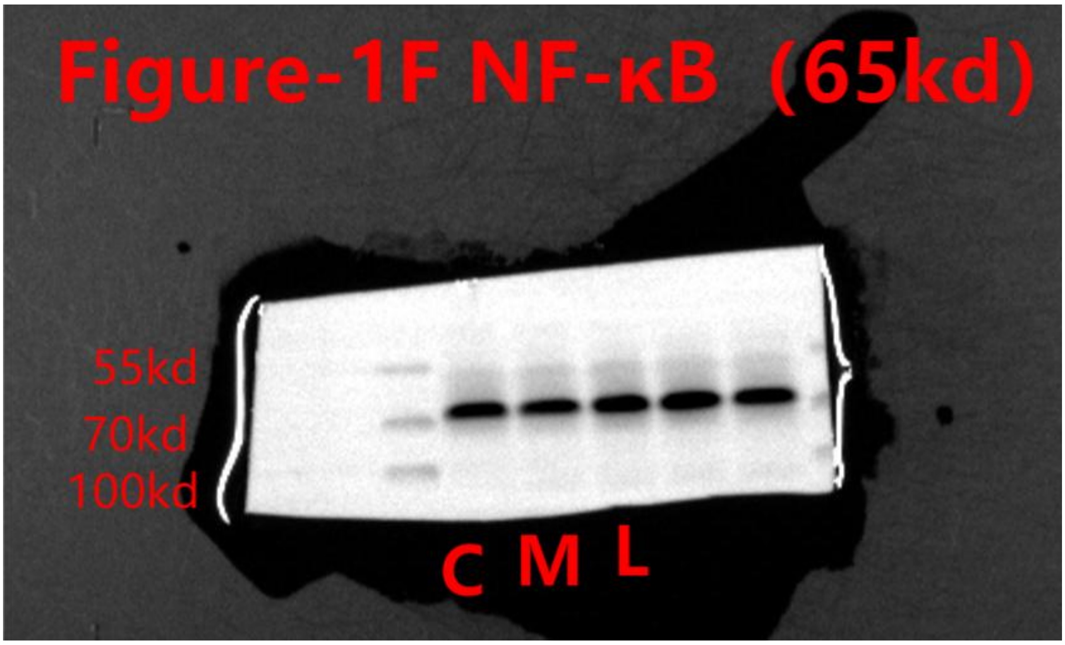
Figure-1F NF-κB (65kd)
55kd
70kd
100kd
C M L

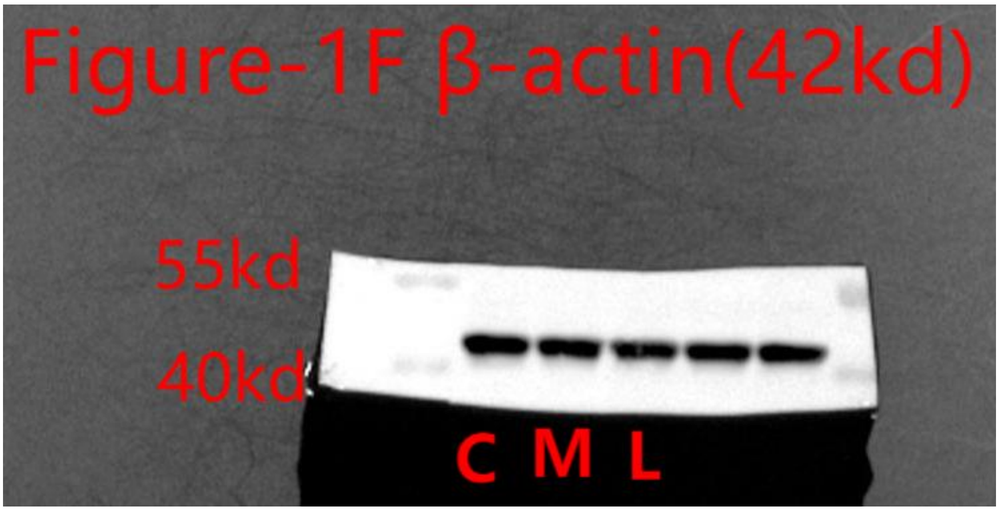
Figure-1F β-actin(42kd)
55kd
40kd
C M L

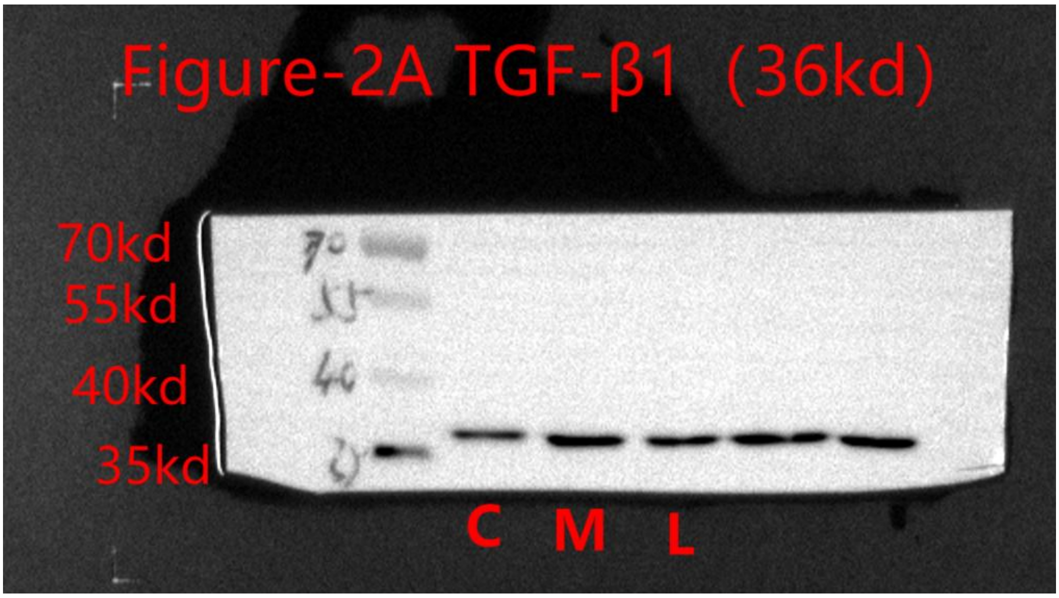
Figure-2A TGF-β1 (36kd)
70kd
55kd
40kd
35kd
C M L

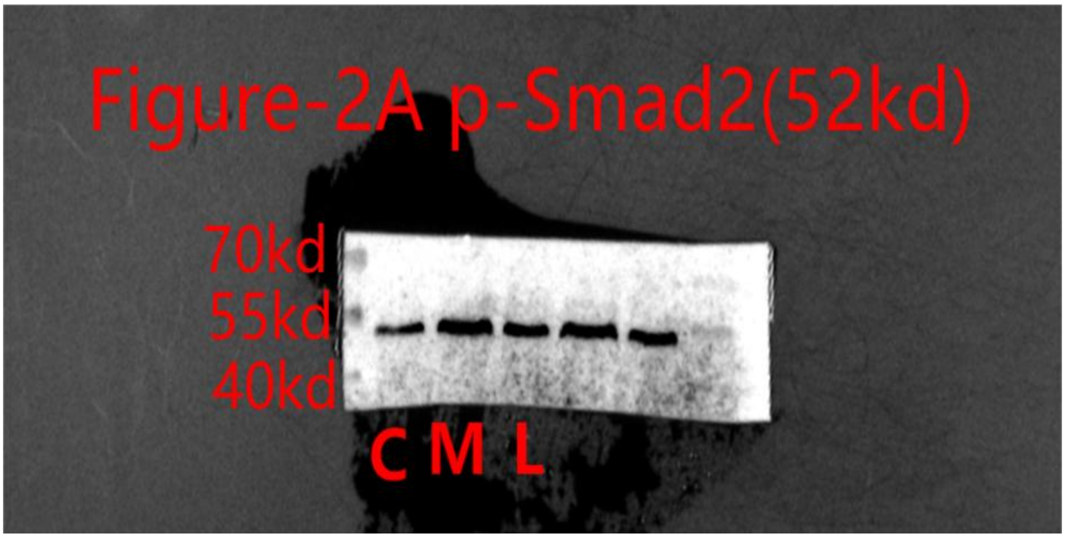
Figure-2A p-Smad2(52kd)
70kd
55kd
40kd
C M L

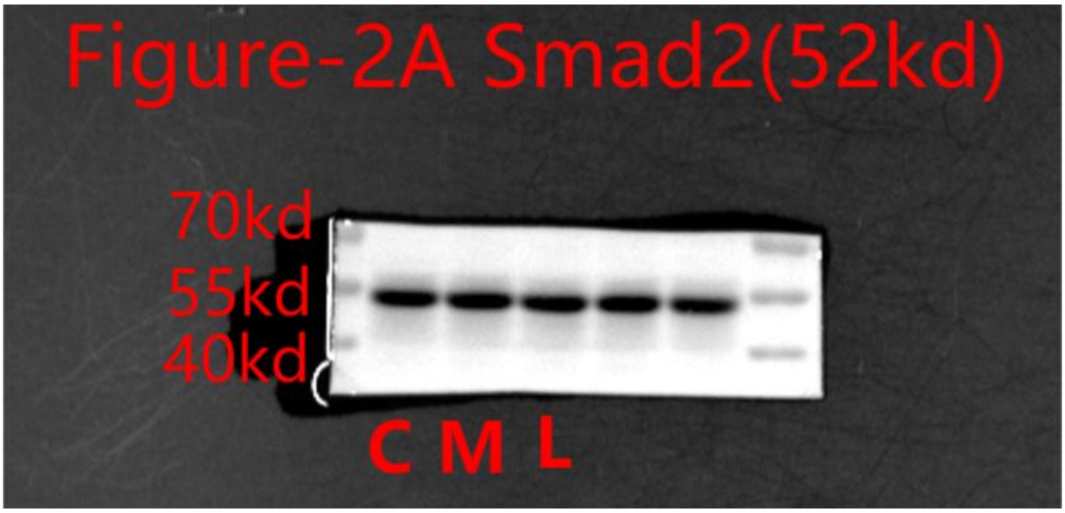
Figure-2A Smad2(52kd)
70kd
55kd
40kd
C M L

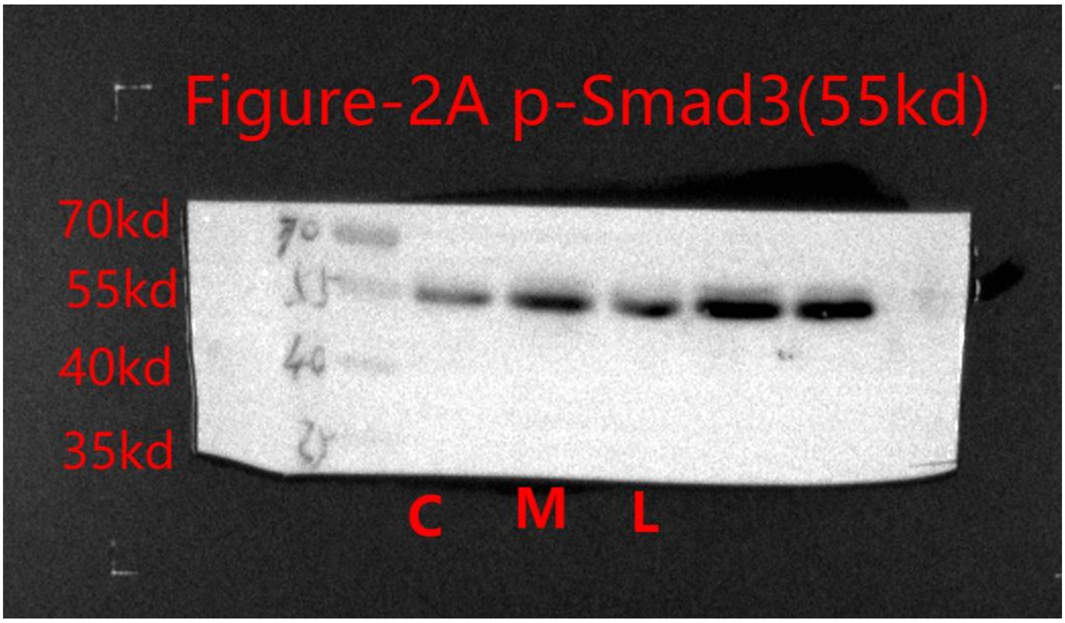
Figure-2A p-Smad3(55kd)
70kd
55kd
40kd
35kd
C M L

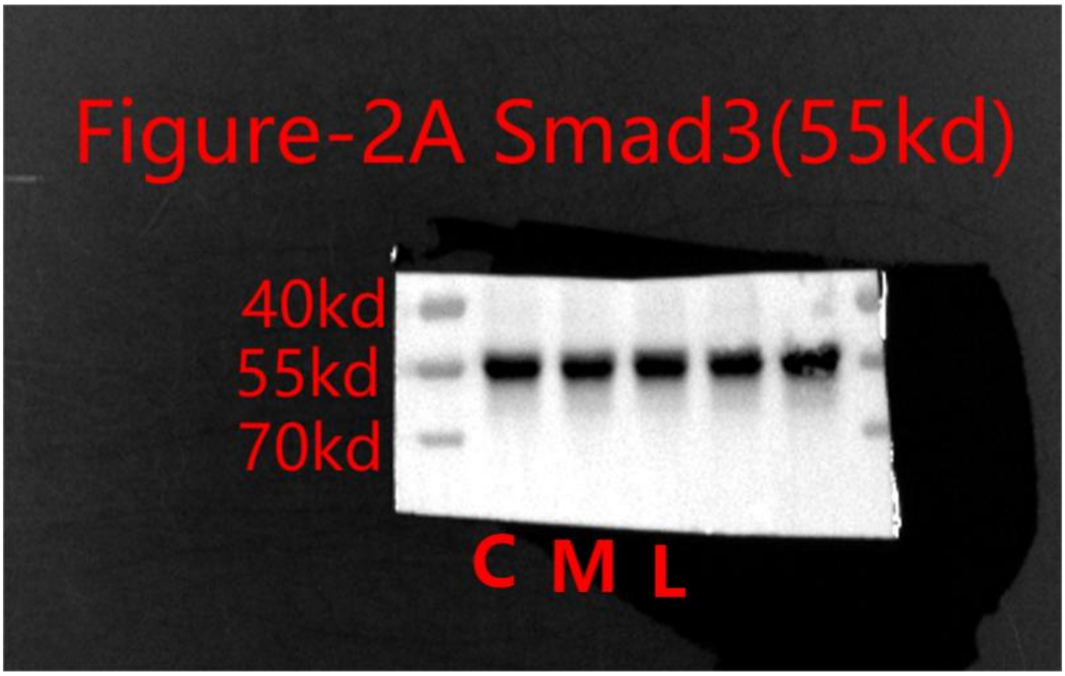
Figure-2A Smad3(55kd)
40kd
55kd
70kd
C M L

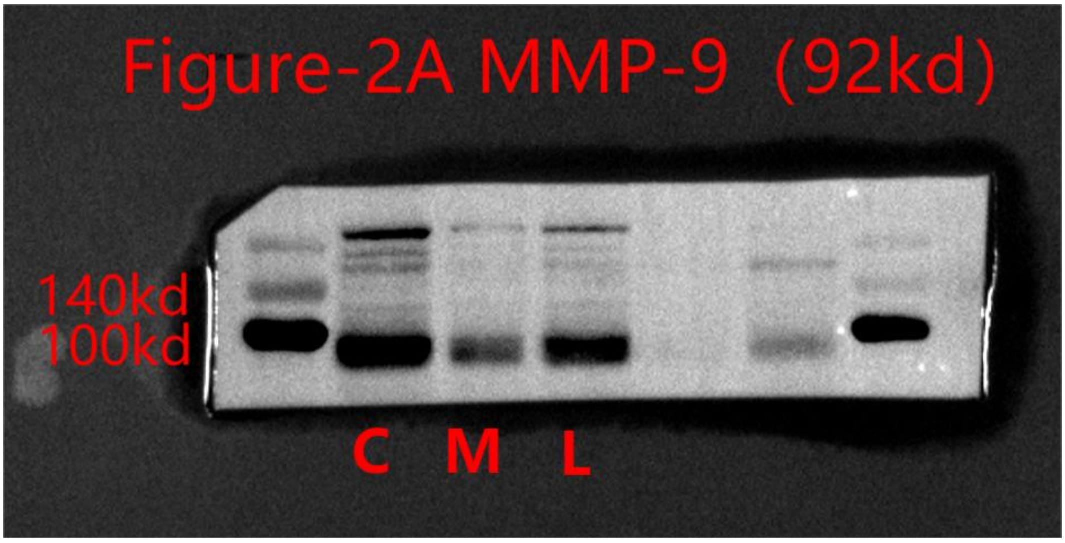
Figure-2A MMP-9 (92kd)
140kd
100kd
C M L

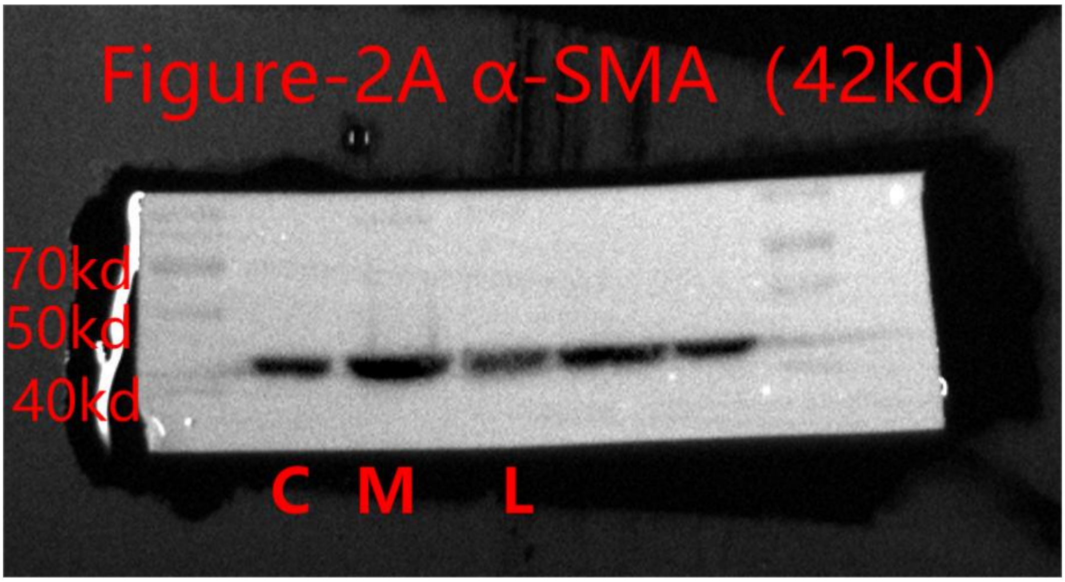
Figure-2A α-SMA (42kd)
70kd
50kd
40kd
C M L

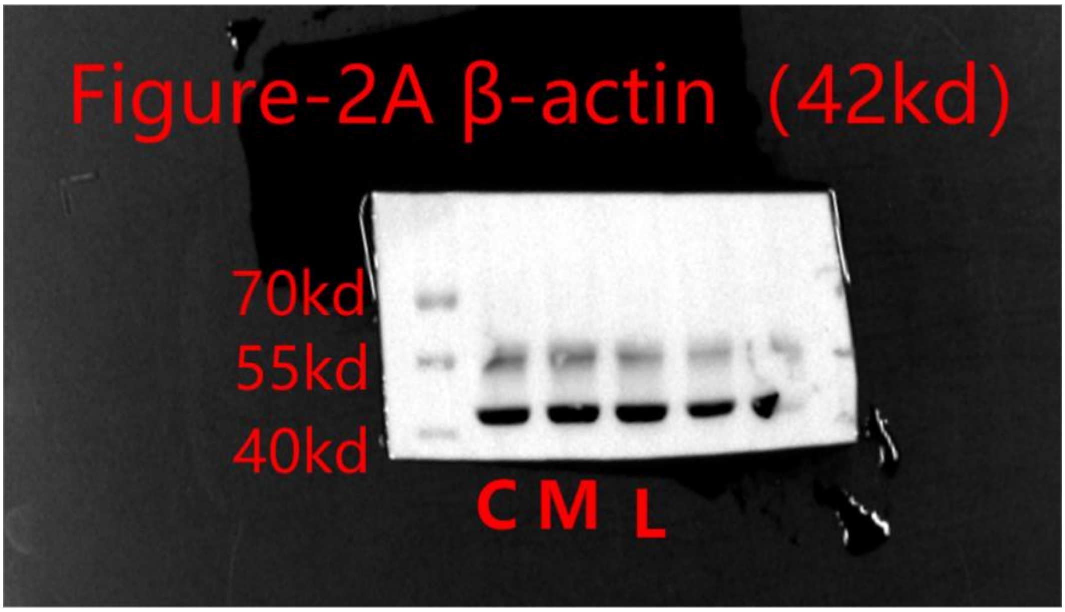
Figure-2A β-actin (42kd)
70kd
55kd
40kd
C M L
